# Effect of Climate and Land Use on the Spatio-Temporal Variability of Tick-Borne Bacteria in Europe

**DOI:** 10.3390/ijerph15040732

**Published:** 2018-04-12

**Authors:** Roberto Rosà, Veronica Andreo, Valentina Tagliapietra, Ivana Baráková, Daniele Arnoldi, Heidi Christine Hauffe, Mattia Manica, Fausta Rosso, Lucia Blaňarová, Martin Bona, Marketa Derdáková, Zuzana Hamšíková, Maria Kazimírová, Jasna Kraljik, Elena Kocianová, Lenka Mahríková, Lenka Minichová, Ladislav Mošanský, Mirko Slovák, Michal Stanko, Eva Špitalská, Els Ducheyne, Markus Neteler, Zdenek Hubálek, Ivo Rudolf, Kristyna Venclikova, Cornelia Silaghi, Evelyn Overzier, Robert Farkas, Gábor Földvári, Sándor Hornok, Nóra Takács, Annapaola Rizzoli

**Affiliations:** 1 Department of Biodiversity and Molecular Ecology, Research and Innovation Centre, Fondazione Edmund Mach, 38010 San Michele all’Adige, Italy; roberto.rosa@fmach.it (R.R.); veroandreo@gmail.com (V.A.); iva.barakova@gmail.com (I.B.); daniele.arnoldi@fmach.it (D.A.); heidi.hauffe@fmach.it (H.C.H.); mattia.manica@fmach.it (M.M.); fausta.rosso@fmach.it (F.R.); annapaola.rizzoli@fmach.it (A.R.); 2 Department of Earth Observation Science, Faculty of Geo-Information Science and Earth Observation (ITC), University of Twente, 7500 AE Enschede, The Netherlands; 3 Institute of Zoology, Slovak Academy of Sciences, 84506 Bratislava, Slovakia; marketa.derdakova@gmail.com (M.D.); svitalkova@gmail.com (Z.H.); uzaemkaz@savba.sk (M.K.); jasna.kraljik@savba.sk (J.K.); lenka.mydlova@gmail.com (L.M.); slovak.mirko@gmail.com (M.S.); 4 Parasitological Institute, Slovak Academy of Sciences, 04001 Košice, Slovakia; blanarova@saske.sk (L.B.); mosansky@saske.sk (L.M.); stankom@saske.sk (M.S.); 5 Department of Anatomy, Pavol Jozef Šafárik University, 04001 Košice, Slovakia; martinbonask@gmail.com; 6 Institute of Virology, Biomedical Research Center, Slovak Academy of Sciences, 84505 Bratislava, Slovakia; ela.kocianova@gmail.com (E.K.); lenka.berthova@gmail.com (L.M.); eva.spitalska@savba.sk (E.Š.); 7 Avia-GIS, Risschotlei 33, 2980 Zoersel, Belgium; educheyne@avia-gis.com; 8 Mundialis GmbH & Co. KG, 53111 Bonn, Germany; neteler@mundialis.de; 9 Institute of Vertebrate Biology, v.v.i., Academy of Sciences of the Czech Republic, 60365 Brno, Czech Republic; zhubalek@brno.cas.cz (Z.H.); rudolf@ivb.cz (I.R.); venclikova@imc.cas.cz (K.V.); 10 Institute of Macromolecular Chemistry CAS, 16206 Prague 6, Czech Republic; 11 Comparative Tropical Medicine and Parasitology, Ludwig-Maximilians-Universität, 80802 Munich, Germany; cornelia.silaghi@fli.de (C.S.); EvelynOverzier@gmx.de (E.O.); 12 Institute of Parasitology, National Centre for Vector Entomology, Vetsuisse-Faculty, University of Zurich, 8057 Zürich, Switzerland; 13 Institute of Infectology, Friedrich-Loeffler-Institut, 17493 Greifswald, Germany; 14 Department of Parasitology and Zoology, University of Veterinary Medicine, 1078 Budapest, Hungary; farkas.robert@univet.hu (R.F.); foldvarigabor@gmx.de (G.F.); hornok.sandor@univet.hu (S.H.); takacs.nora@univet.hu (N.T.)

**Keywords:** land use, acarological hazard, *Borrelia burgdorferi* sensu lato, *Anaplasma phagocytophilum*, *Rickettsia* spp., normalized difference vegetation index, density of infected nymphs

## Abstract

The incidence of tick-borne diseases caused by *Borrelia burgdorferi* sensu lato, *Anaplasma phagocytophilum* and *Rickettsia* spp. has been rising in Europe in recent decades. Early pre-assessment of acarological hazard still represents a complex challenge. The aim of this study was to model *Ixodes ricinus* questing nymph density and its infection rate with *B. burgdorferi* s.l., *A. phagocytophilum* and *Rickettsia* spp. in five European countries (Italy, Germany, Czech Republic, Slovakia, Hungary) in various land cover types differing in use and anthropisation (agricultural, urban and natural) with climatic and environmental factors (Normalized Difference Vegetation Index (NDVI), Normalized Difference Water Index (NDWI), Land Surface Temperature (LST) and precipitation). We show that the relative abundance of questing nymphs was significantly associated with climatic conditions, such as higher values of NDVI recorded in the sampling period, while no differences were observed among land use categories. However, the density of infected nymphs (DIN) also depended on the pathogen considered and land use. These results contribute to a better understanding of the variation in acarological hazard for *Ixodes ricinus* transmitted pathogens in Central Europe and provide the basis for more focused ecological studies aimed at assessing the effect of land use in different sites on tick–host pathogens interaction.

## 1. Introduction

Human alteration of natural ecosystems is now evident on more of 75% of the Earth’s ice-free land mass as a result of urbanization, agricultural and other land uses, with less than a quarter remaining as intact habitats [1]. Urbanization, in particular, has increased worldwide in recent decades and more than half of the world’s population now lives in urban areas, with the expectation that 66% will live in urban areas by 2050 [2,3].

Human land use and exploitation of natural ecosystems represent one of the major drivers of zoonotic disease emergence by disrupting disease dynamics and cross-species transmission in multi-host, multi-pathogen systems (‘perturbation hypothesis’) and/or by increasing exposure of hosts to novel pathogens (‘novel pathogen pool hypothesis’) [4]. However, understanding the mechanisms by which land use change leads to disease emergence is still rudimentary.

Vector borne diseases, and in particular those transmitted by ticks, appear to be particularly sensitive to land use changes [5] and, therefore, they represent a valuable model to quantify the indirect impact of anthropization and land use changes on human and animal health.

The widespread occurrence of the castor bean tick, *Ixodes ricinus* in Europe, and the pathogens it transmits, together with the rise of clinical cases of tick-borne diseases (TBDs) in humans and livestock, have made TBDs one of the major One Health issues in recent years [6]. *I. ricinus* has a broad ecological plasticity and capacity to exploit anthropic landscapes, and its distribution has increased in the last three decades as a result of more favorable biotic and abiotic conditions [5]. Concomitantly, the annual incidences of bacterial diseases such as Lyme borreliosis and rickettsiosis have also increased steadily [7,8]. Several authors have suggested that the prediction of ‘acarological hazard’ in different habitat conditions could be useful to public health authorities for planning and implementing targeted educational and preventive actions for TBDs [9,10]. Hazard is defined in epidemiological risk assessment procedures as “the set of circumstances that could lead to harm” [11], i.e., in this case, the occurrence within natural enzootic cycles of certain tick-borne pathogens (TPBs) with known pathogenic potential. In turn, “risk is the actual exposure of susceptible hosts to TBPs” [11] that takes into account the interaction between infected ticks and humans [11]. The acarological hazard depends on the co-occurrence of TBPs, a competent and active vector (e.g., *Ixodes ricinus*) and a competent reservoir host enabling the transmission in endemic cycles of the pathogens from infected to uninfected ticks [12].

Predicting acarological hazard is challenging and, despite significant progress in estimating tick occurrence and abundance, the approximation of infection rates in ticks is more complex, since this is related to a series of parameters that are difficult to measure in the field: tick abundance and tick seasonal activity, as well as the contact rate of infected ticks with reservoir hosts (where reservoir capacity varies according to vertebrate species and their immune status) [13].

The seasonal abundance of questing *I. ricinus* nymphs (considered the most relevant epidemiological developmental stage) and their questing activity pattern are dependent on habitat structure, microclimatic conditions, and the availability of tick-feeding hosts [14,15]. The prediction of spatial and temporal distribution of *I. ricinus* based on ecological and climatic factors has progressed from studies capturing the short term phenology of the ticks based on ground climate data with simple models [16] to more complex ones based on remote sensing data using correlative [16,17] or modified matrix [18] approaches. Remote sensing (RS) imagery has also proven to be very useful in predicting changes in habitat and climatic conditions at different temporal and spatial scales, and it has been widely used to map the distribution of several disease vectors [19], including *I. ricinus* [20,21,22].

Here, we attempt to assess the spatio-temporal variability of *B. burgdorferi* s.l., *A. phagocytophilum* and spotted fever group (SFG) *Rickettsia* spp. infection in questing *I. ricinus* nymphs. Specifically, we tested the associations between the abundance of host-seeking *I. ricinus* nymphs and the density of infected nymphs (DIN) with a combination of RS parameters such as Land Surface Temperature (LST), Normalized Difference Vegetation Index (NDVI), Normalized Difference Water Index (NDWI), and climatic data obtained from interpolations of meteorological station data, such as accumulated precipitation. To fit our models, we used multi-year field data on the abundance of questing *I. ricinus* and prevalence of infection with TBPs from five EU countries. Our aim was to provide a first insight on the variables affecting TBDs infection hazard on a wide geographical scale in sites with different land use.

## 2. Materials and Methods

### 2.1. Questing Tick Data

**Figure 1:**
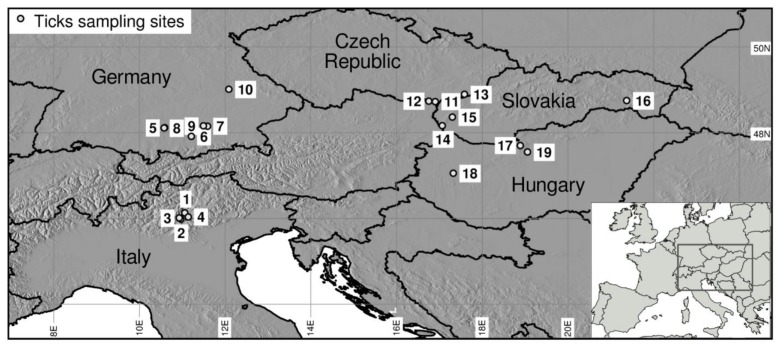
Map of the 19 ticks sampling sites in Italy, Germany, Czech Republic, Slovakia and Hungary (see Table 1).

Questing ticks were collected monthly during their peak activity in spring (April, May, June) from 2011 to 2013 from 19 sampling sites distributed across Italy, Germany, Slovakia, Czech Republic and Hungary within the framework of the EU FP7 project EDENext (Figure 1).

In each country, urban, agricultural and natural sites were sampled (Table 1), defined according to CORINE land cover classification layers with a spatial resolution of 100 m [23]. 

Specifically, urban sites belonged to category 1.4.1 i.e., green urban areas (these are areas with vegetation within an urban context, ornamental and recreational character and public accessibility), agricultural sites belonged to category 2.3.1 i.e., pastures (here are included areas with permanent grassland), natural sites belonged to category 3.1.3 i.e., mixed forests (in these forests, shrub and bush understorey is present and neither conifer or deciduous species predominate).

Questing ticks were sampled using the standard dragging method. A 1 m^2^ white blanket attached to a rod was pulled over the vegetation along three established transects of 100 m^2^ (i.e., 0.01 ha) per sampling site, covering an area of 300 m^2^. The blanket was checked for the presence of ticks on both sides every 5 m. Ticks collected were put in vials, separately per transect, and brought to the laboratory for identification and analyses. Although the dragging method preferentially captures *I. ricinus* nymphs, underestimating the true tick abundance in the area and introducing a bias with regard to instar composition (Mejlon et al., 1997), the goal in this study was to estimate temporal and spatial variation of questing nymph abundance, so larval and adult stages were not included in the analyses. All captured ticks were identified to species and life stage using a stereo-microscope and reference keys [25,26,27,28]. Nymphs were individually placed in vials with 70% ethanol and stored at −20 °C until DNA extraction. 

*Ixodes ricinus* nymph counts were pooled over the sampling periods, and questing nymph density (nymphs/hectare) for each year and site was computed. To assess the relative risk to public health, the estimated DIN per hectare for each pathogen was calculated by multiplying the infection prevalence for each pathogen (%) by the density of questing nymphs for each sampling site.

### 2.2. Molecular Analyses

Each partner laboratory screening of TBPs was performed by PCR-based methods with previously published primers: in Italy, *Borrelia* spp., *A. phagocytophilum* and *Rickettsia* spp. were detected according to Rijpkema et al. [29], Massung et al. [30], and Reye et al. [31], respectively, with minor modifications (see [32]). In Germany and Slovakia, screening for *Borrelia* spp. was carried out according to Derdáková et al. [33]. In Germany, Slovakia, Czech Republic and Hungary, detection of *A. phagocytophilum* and *Rickettsia* spp. was carried out according to Courtney et al. [34] and Regnery et al. [35], respectively; and described, for Germany in Overzier et al. [36,37] for Slovakia in Blaňarová et al. [38], Svitálková et al. [39], Špitalská et al. [40] and Minichová et al. [41]; for the Czech Republic in Venclíková et al. [42]; and for Hungary in Hornok et al. [43]. The comparability of results by each partner laboratory was guaranteed by an inter-laboratory quality ring test of molecular PCR methods for the detection of *I. ricinus* transmitted pathogens. Analyses for species/genospecies identification of each pathogen were not carried out by all partner laboratories.

### 2.3. Climatic and Environmental Data

Environmental predictors were selected based on published evidence of their importance to tick populations [22,44,45,46]. For each sampling site, we obtained climatic and environmental data from RS and interpolated climatic datasets. All environmental data were processed in GRASS GIS 7 [47], and extracted from the spatial database at the locations corresponding to the sampling sites. Sampling transect lengths are below the pixel size for most spatial data, so data averaging over a wider area was not considered appropriate.

Land surface temperature (LST) data were obtained from EuroLST dataset [48]. These data were collated from the Moderate Resolution Imaging Spectroradiometer (MODIS) products MOD11A1 and MYD11A1. The original MODIS LST products were reconstructed (i.e., gap-filled to remove void pixels due to clouds) and downscaled from 1000 to 250 m resolution, a higher resolution Digital Elevation Model (DEM) [48]. For the analyses, daily reconstructed LST data were used to derive monthly and seasonal mean temperature (3-month moving windows) for the current (*t*) and previous year (*t* − 1). This 3-month aggregation has already proven to perform better as a predictor when compared with shorter time windows [49]. We also obtained autumnal cooling [50] as the slope of a linear regression between daily LST and DOY (day of year) over three months (August, September and October) [50]. Autumnal cooling is by definition only estimated for the year before each sampling period, while the other variables are estimated both for the sampling year and the year before. Spring warming, on the other hand, was estimated as the slope of a linear regression between daily LST and DOY (day of year) over February, March and April of the current and previous year.

Precipitation was measured as total rainfall and number of days with rainfall per month, and in a 3-month moving window as described above, from the gridded ECA&D dataset (European Climate Assessment & Dataset, Version 13.1 [51]) at an approximately 25 km pixel resolution [52].

We obtained the NDVI from MOD13Q1, and the NDWI from MOD09A1. Both are MODIS composite products (16 days and eight days, respectively) and have a spatial resolution of 500 m. The gaps in NDVI and NDWI time series were filled and outliers removed using a harmonic analysis of each series [53]. These variables were used as proxies for vegetation coverage (NDVI) and for available environmental water (NDWI), which includes surface water as well as vegetation water content. We also aggregated NDVI and NDWI data per month and seasonally (3-month moving window from February to August) for the current year (year of sampling) and the year before samplings using the arithmetic average. January NDVI and NDWI were not included in the analyses due to the presence of snow cover, which can dramatically alter the reliability of satellite acquisition of these parameters [50]. Spring greening and spring wetting were estimated as the slope of a linear regression between NDVI or NDWI, respectively, and DOY over three months (February, March and April) for the sampling and previous year.

### 2.4. Statistical Analyses

We investigated the relationship between nymph density and DIN with a categorical variable representing the land use category (agricultural, natural, urban) and several environmental and climatic variables (i.e., LST, precipitation, NDVI and NDWI). All statistical analyses were performed using R version 3.4.3 [54].

The code used for statistical analysis is available at [55].

Negative Binomial Generalized Linear Mixed Models (GLMM) (through the glmmTMB package, [56]) were used to investigate the association between nymph density and land use category accounting for environmental and climatic variables. Negative Binomial distribution was chosen to account for overdispersion in the count data. DIN were modeled using Linear Mixed Models (LMM) after log-transforming the DIN (through the lme4 package; [57]). In both analyses, country and year were considered as random effects. All climatic and environmental variables were standardized (i.e., by subtracting their mean and dividing by their standard deviation) before including them in the models. The use of standardized variables was recommended as some explanatory variables, such as total precipitation, had a much larger range of variation compared to the rest of the variables considered.

For all climatic and environmental variables, a preliminary analysis was carried out to ascertain in which period they proved to be the best predictor of both total nymphs counted and DIN. Precisely, for each environmental variable, univariate models with a single covariate (environmental variable) in a specific temporal window were computed in turn. We considered fifteen different temporal windows that were: (i) each of the six months from January to June during the year of collection (as no tick collections were carried out after June); (ii) four wider windows obtained by aggregating a 3-month period from January to June of the same year (i.e., January–February–March, February–March–April, March–April–May, April–May–June); (iii) each quarter of the previous year; and (iv) the whole previous year. Mean values were considered for each variable except for precipitation where the total over each temporal window was computed.

For each variable (LST, precipitation, NDVI and NDWI), the temporal window producing the lowest AIC (Akaike Information Criterion) was selected for inclusion in a subsequent full model. Terms that were not significant for any of the temporal windows were not included in the full model. The Variance Inflation Factor (VIF) was used to test for collinearity among all explanatory variables in the full model, removing variables with VIF > 4 [58]. Following exclusion of collinear and non-significant variables, we developed full models for both nymph density and DIN including the remaining environmental and climatic variables, each measured over its optimum temporal window as selected through preliminary analyses, along with the habitat type. Starting from the initial full model, we carried out a model selection procedure (based on AIC) to find the best model for nymph density and DIN for three different pathogens. Residuals of the best models (lowest AIC) were used to check for model assumptions. For all models, we computed the variance explained (conditional R^2^) following [59].

## 3. Results

### 3.1. Questing Nymph Abundance

**Figure 2:**
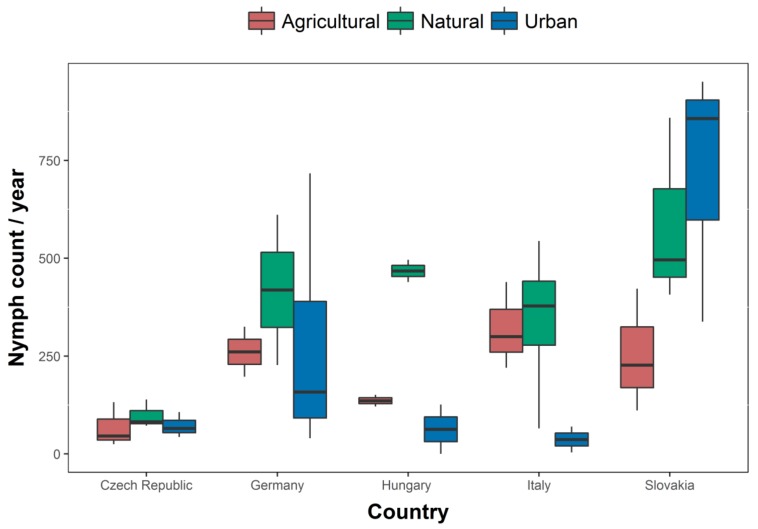
Boxplot of observed questing *I. ricinus* nymphs collected over the year (April–May–June period) in different countries and habitat types; *x*-axis = country; *y*-axis = number of collected nymphs.

In total, 17,832 ticks were collected of which 13,291 were nymphs. The highest value for questing nymph density per country and per year (April–May–June period) was observed in Slovakia (average value = 518.7 nymph/year/300 m^2^ (3 transects of 100 m^2^ per site); 95% confidence interval = 352.1–764), while the lowest was recorded in the Czech Republic (79; 57.1–109.3) (Figure 2). The remaining countries showed similar values (Italy: 266.4, 140–506.9; Germany: average = 278.7, 190.8–407.2; Hungary: 222.2, 210.6–234.4). The highest value for questing nymph density per land use per year (April–May–June period) was observed within natural sites (370.9, 256.4–536.5), compared to the urban sites (256.3, 153.5–428) and agricultural ones (208.9, 144.2–302.6) (Figure 2). However, as shown by the following analysis, these differences were not statistically significant.

**Figure 3:**
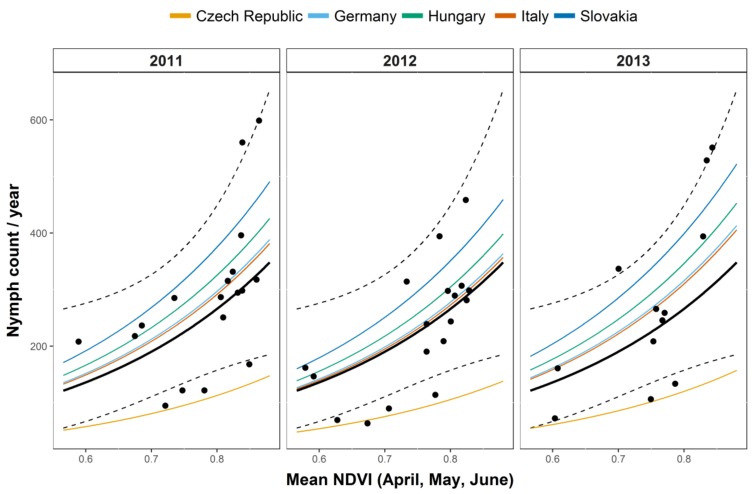
Best models for questing nymph density (Negative Binomial Generalized Linear Mixed Model); values on the *x*-axis represent the mean normalized difference vegetation index (NDVI) over three months (April, May, June); values on the *y*-axis represent the number of collected nymphs. The solid black line represents the fitted values (highlighting the relationship between NDVI and the typical country-year) computed by considering the accumulated precipitation in the 4th quarter at its mean value. Dashed lines are the 95% confidence intervals for the fitted values. Coloured lines represent the association between the number of collected nymphs and NDVI within each country. Points are observed values.

The results of the Negative Binomial GLMM indicate that questing nymph density was significantly associated with environmental and climatic variables, while no differences were observed among land use categories (Table 2). The best model explaining questing nymph density included a positive effect of average NDVI computed in the period April–May–June (Figure 3), the same period in which sampling was carried out, and a non-significant negative effect of the accumulated precipitation in the last quarter of the previous year (Table 2). The model for questing nymph explained the 32% of the variance (R^2^ = 0.32).

### 3.2. Infection Prevalence and DIN

The prevalence of infection with *A. phagocytophilum* and *Rickettsia* spp. was assessed for all countries, with values 2.51% (se = 0.32%) and 7.2% (se = 0.65%), respectively. *B. burgdorferi* s.l. (screened in Italy, Germany and Slovakia) was the pathogen with the highest prevalence of infection, with an overall prevalence of 19.32% (se = 1.42%). 

**Figure 4:**
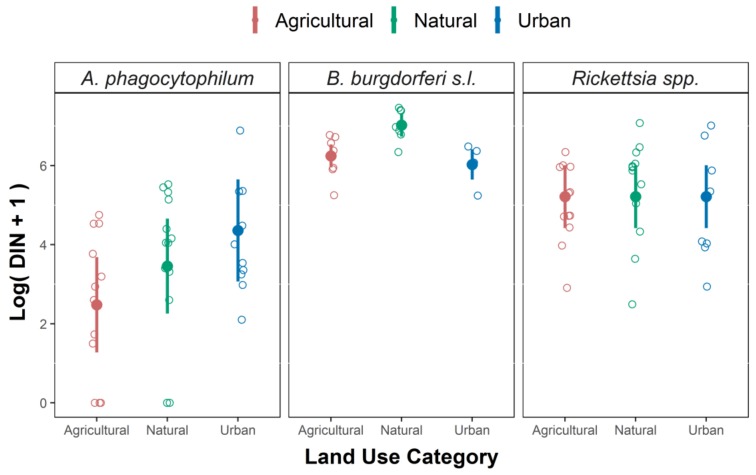
Best Linear Mixed Models for density of infected nymphs (DIN) for *A. phagocytophilum* (**left panel**), *B. burgdorferi* s.l. (**central panel**), *Rickettsia* spp. (**right panel**). Filled circles represent the fitted values, and empty circles are the observed values. Solid lines represent the 95% confidence intervals for the fitted values.

As described within the methods, to assess the risk to public health we carried out the statistical analysis for the DIN, obtained by multiplying the infection prevalence for each pathogen by the density of questing nymphs for each sampling site. LMM models indicate that DIN varied significantly among land use categories for *A. phagocytophilum* and *B. burgdorferi* while no differences were observed in DIN for *Rickettsia* spp. (Table 3, Figure 4). The model for DIN with *A. phagocytophilum* explained the 59% of the variance (R^2^ = 0.59), while R^2^ for models for DIN with *B. burgdorferi* s.l. and *Rickettsia* spp. were 0.68 and 0.66 respectively.

Specifically, the natural habitat had the highest DIN values for *B. burgdorferi* s.l., the urban habitat type showed the highest DIN values for *A. phagocytophilum*, while, for *Rickettsia* spp., no differences were observed among habitat types (Figure 4).

Concerning the effect of environmental variables on DIN, a negative effect of the accumulated precipitation during the first three months (Jan–Feb–Mar) of the previous year was observed on DIN for *B. burgdorferi* s.l. (Table 3). On the other hand, the best model for *A. phagocytophilum* and *Rickettsia* spp. included a positive effect of NDVI in the same year. Precisely, NDVI recorded in January was positively related to DIN for *A. phagocytophilum* and NDVI recorded in March was positively related to DIN for *Rickettsia* spp. (Table 3). 

## 4. Discussion

Land use change is considered among the most important drivers of TBD emergence. Therefore, in a given area, shifting levels of anthropisation, type of socio-economical activity, and changes in the presence of domestic and wildlife species can also provoke the conditions promoting variation in the acarological hazard for TBPs. Predicting tick population seasonal dynamics together with their pathogen infection rate is useful for an early pre-assessment of the acarological hazard, but the parameters are challenging to estimate. In this study, we used environmental and climatic variables that are known to be related to *I. ricinus* phenology and can be obtained on a wide geographical scale from RS imagery and different types of land use, to explain the spatio-temporal variability in questing *I. ricinus* nymphs spring density as well as their pathogen infection rates among different European countries.

NDVI is a controversial predictor of tick abundance: some authors consider its relationship with vegetation water content arguable [46], and do not consider NDVI a suitable predictor of tick abundance [60]. Others confirm the correlation of NDVI with relative humidity in the vegetation layer [61], and therefore, with *I. ricinus* survival rate and tick abundance and questing behavior [15,22,36,37,62,63,64]. In our study, NDVI recorded in late spring of the sampling year was a very good predictor of questing tick density, supporting the latter hypothesis.

Moreover, it has been suggested that RS of vegetation moisture (e.g., NDWI) may outperform NDVI in modelling the incidence of Lyme borreliosis [46]. In our analyses, however, we did not find any significant association between questing nymph density or DIN with NDWI, with the exception of a marginally significant positive association of March NDWI with *Rickettsia* spp. DIN in univariate models (results not shown). NDWI is based on canopy reflectance values, which could explain why this parameter fails to predict conditions on the forest floor that are more closely correlated with the occurrence of ticks [62].

NDVI was also positively correlated with DIN of *A. phagocytophilum* and *Rickettsia* spp. when measured a few months before or partially overlapping with the tick sampling period, making it a suitable early predictor of annual acarological hazard within a reasonable time frame for these pathogens.

With regards to DIN, we found that the accumulated rainfall in the first trimester of the previous year had a negative effect on the DIN of *B. burgdorferi* s.l., while no correlation was detected for the other pathogens studied here. The negative effect of rainfall detected for DIN of *B. burgdorferi* s.l. was found also in the nymphs abundance model. Therefore, one can speculate that the driving effect of rainfall on DIN is probably driven by the effect of rainfall on nymph abundance. This association may be related to the effect of rainfall on tick oviposition rates [65], number of eggs per clutch and/or hatching success rather than on questing activity [66], since heavy rain silts up egg masses [67]. Therefore, some climatic factors during peak months of egg deposition could be critical for subsequent distribution of ticks. The effect of land use category on the DIN was different according to the pathogen considered. DIN of *B. burgdorferi* s.l. was higher in natural habitats, probably because of the occurrence and abundance of several competent reservoirs for these bacteria, such as rodents and wild birds. Some studies suggest that free-living ungulates, which are not competent reservoir hosts for *B. burgdorferi* s.l., might act as dilutors, i.e., they reduce the prevalence of this pathogen in ticks [68]. However, it is controversial whether the dilution effect occurs regardless of host species ratio or only at unrealistically high densities of deer [69]. Anyway, in order to fully understand the effect of vertebrate host species composition in driving infection prevalence, detailed data on pathogen species/strain identification would be needed. 

Regarding *A. phagocytophilum*, the highest DIN was detected in urban sites with a particularly high value in Slovakia. In this site, the high roe deer density of ca. 300/1000 ha [70] is believed to support the maintenance of high density of questing nymphs. The overall highest DIN for *A. phagocytophilum* in urban areas may also be attributed to the common occurrence of hedgehogs: in a previous study, it was observed that 76.1% of *Erinaceus roumanicus* were infected with this pathogen in a city park of Budapest [71]. The role of urban parks, in particular when their size is relevant and/or their connectivity with natural surrounding areas is possible, is to favor the interaction among vectors, wildlife (including those with large size), domestic animals and humans. For this reason, they are becoming emerging hotspots for vector borne pathogens transmission in Europe [72].

Due to the efficient transovarial transmission of the SFG *Rickettsia* spp., *I. ricinus* is thought to be both their main vector and reservoir [73]. DIN with *Rickettsia* spp. seems to be proportional to density of host-seeking nymphs in the studied countries, with significantly higher values in Germany and Slovakia, while no differences were found between habitat types. The role of vertebrate hosts in *Rickettsia* transmission remains to be elucidated.

## 5. Conclusions

In conclusion, our study provides evidence of variation of the acarological hazard for *I. ricinus* transmitted pathogens in relation to habitat type and climatic condition. These latter, expressed in NDVI, measured in late spring, could be used as a good predictor of questing nymphs density. On the other hand, DIN shows a complex relationship with climatic and land use variables according to the pathogens considered, demonstrating the need to perform more detailed ecological studies aimed at assessing the effect of land use in different habitat types on TBP emergence. 

## Figures and Tables

**Table 1:** Description of study sites (see also Figure 1). Elevations were taken from Global Multi-Resolution Terrain Elevation Data 2010 (mn30_grd layer [24]).

Country	Site Number	Sampling Site	Land Use Category	Altitude (m a.s.l.)	Latitude	Longitude
Italy	1	Lamar	Natural	784	46.128726	11.058944
Italy	2	Cavedine	Agricultural	717	45.985402	10.963142
Italy	3	Pietramurata	Natural	468	46.013258	10.927981
Italy	4	Trento	Urban	285	46.035187	11.139236
Germany	5	Tussenhausen	Natural	640	48.118279	10.589147
Germany	6	Kerschlach	Agricultural	724	47.917142	11.212342
Germany	7	Englischer Garten	Urban	514	48.150481	11.590053
Germany	8	Berg Starnberg	Urban	659	48.110117	10.575944
Germany	9	Nymphenburger Schlosspark	Urban	522	48.160814	11.492586
Germany	10	Dörnbergpark Regensburg	Urban	345	49.015478	12.085803
Czech Republic	11	Pohansko	Natural	162	48.727133	16.902319
Czech Republic	12	Valtice	Urban	215	48.734911	16.753142
Czech Republic	13	Suchov	Agricultural	426	48.897442	17.581928
Slovakia	14	Bratislava	Urban	184	48.166667	17.066667
Slovakia	15	Fúgeľka	Natural	386	48.366667	17.300000
Slovakia	16	Rozhanovce	Agricultural	280	48.750000	21.366667
Hungary	17	Pilisszentkereszt	Natural	468	47.700833	18.884722
Hungary	18	Csabrendek	Agricultural	159	47.053889	17.323333
Hungary	19	Budapest	Urban	105	47.550278	19.052778

**Table 2:** Best model for questing nymph density (Negative Binomial Generalized Linear Mixed Model). The columns report the estimated coefficients for explanatory variables, their standard errors, *z*-values (estimate to standard error ratio) and *p*-value for z-statistic. Independent variables have been standardized. NDVI = normalized difference vegetation index.

Explanatory Variable	Estimate	Std. Error	*z*-Value	Pr(>|*z*|)
Intercept	5.457	0.260	21.026	<0.001 ***
NDVI (Apr–May–Jun)	0.264	0.124	2.131	0.033 *
Accumulated precipitation (Oct–Nov–Dec, previous year)	−0.265	0.149	−1.779	0.075

Signif. codes: *** < 0.001; * < 0.05.

**Table 3:** Best parsimonious models for density of infected nymphs (DIN) (Linear Mixed Model) with *A. phagocytophilum*, *B. burgdorferi* s.l. and *Rickettsia* spp. The columns report the estimated coefficients for explanatory variables, their standard errors, *t*-values (estimate to standard error ratio) and *p*-value for the *t*-statistic. Reference level is Agricultural for habitat type.

Model	Explanatory Variable	Estimate	Std. Error	*t* Value	Pr(>|*t*|)
DIN for *Borrelia*	Intercept	6.245	0.148	42.216	<0.001 ***
	Habitat type Natural	0.779	0.209	1.724	0.002 **
	Habitat type Urban	−0.215	0.245	−0.878	0.395
	Accumulated Precipitation (Jan-Feb-Mar, previous year)	−0.310	0.095	−3.268	0.006 **
DIN for *Anaplasma*	Intercept	2.478	0.615	4.026	0.006 **
	Habitat type Natural	0.980	0.540	1.814	0.080
	Habitat type Urban	1.882	0.591	3.185	0.003 **
	NDVI (January)	0.740	0.261	2.831	0.008 **
DIN for *Rickettsia*	Intercept	5.214	0.405	4.267	<0.001 ***
	NDVI (March)	0.488	0.153	29.603	0.003 **

Signif. codes: *** <0.001; ** <0.01.
